# F-18 labelled PSMA-1007: biodistribution, radiation dosimetry and histopathological validation of tumor lesions in prostate cancer patients

**DOI:** 10.1007/s00259-016-3573-4

**Published:** 2016-11-26

**Authors:** Frederik L. Giesel, B. Hadaschik, J. Cardinale, J. Radtke, M. Vinsensia, W. Lehnert, C. Kesch, Y. Tolstov, S. Singer, N. Grabe, S. Duensing, M. Schäfer, O. C. Neels, W. Mier, U. Haberkorn, K. Kopka, C. Kratochwil

**Affiliations:** 1 Department of Nuclear Medicine, University Hospital Heidelberg, INF 400, 69120 Heidelberg, Germany; 2 Department of Urology, University Hospital Heidelberg, Heidelberg, Germany; 3 Division of Radiopharmaceutical Chemistry, German Cancer Research Center (dkfz), Heidelberg, Germany; 4 ABX-CRO, Dresden, Germany; 5 Section of Molecular Urooncology, Department of Urology, Medical Faculty Heidelberg, University Hospital Heidelberg, Heidelberg, Germany; 6 Institute of Pathology, University Hospital Heidelberg, Heidelberg, Germany; 7 Department of Medical Oncology, National Center for Tumor Diseases (NCT), University Hospital Heidelberg, Heidelberg, Germany; 8 Hamamatsu Tissue Imaging and Analysis Center, University of Heidelberg, Heidelberg, Germany

**Keywords:** ^18^F-PSMA, F-18-PSMA, PSMA-1007, PET/CT, Positron emission tomography

## Abstract

**Purpose:**

The prostate-specific membrane antigen (PSMA) targeted positron-emitting-tomography (PET) tracer ^68^Ga-PSMA-11 shows great promise in the detection of prostate cancer. However, ^68^Ga has several shortcomings as a radiolabel including short half-life and non-ideal energies, and this has motivated consideration of ^18^F-labelled analogs. ^18^F-PSMA-1007 was selected among several ^18^F-PSMA-ligand candidate compounds because it demonstrated high labelling yields, outstanding tumor uptake and fast, non-urinary background clearance. Here, we describe the properties of ^18^F-PSMA-1007 in human volunteers and patients.

**Methods:**

Radiation dosimetry of ^18^F-PSMA-1007 was determined in three healthy volunteers who underwent whole-body PET-scans and concomitant blood and urine sampling. Following this, ten patients with high-risk prostate cancer underwent ^18^F-PSMA-1007 PET/CT (1 h and 3 h p.i.) and normal organ biodistribution and tumor uptakes were examined. Eight patients underwent prostatectomy with extended pelvic lymphadenectomy. Uptake in intra-prostatic lesions and lymph node metastases were correlated with final histopathology, including PSMA immunostaining.

**Results:**

With an effective dose of approximately 4.4–5.5 mSv per 200–250 MBq examination, ^18^F-PSMA-1007 behaves similar to other PSMA-PET agents as well as to other ^18^F-labelled PET-tracers. In comparison to other PSMA-targeting PET-tracers, ^18^F-PSMA-1007 has reduced urinary clearance enabling excellent assessment of the prostate. Similar to ^18^F-DCFPyL and with slightly slower clearance kinetics than PSMA-11, favorable tumor-to-background ratios are observed 2–3 h after injection. In eight patients, diagnostic findings were successfully validated by histopathology. ^18^F-PSMA-1007 PET/CT detected 18 of 19 lymph node metastases in the pelvis, including nodes as small as 1 mm in diameter.

**Conclusion:**

^18^F-PSMA-1007 performs at least comparably to ^68^Ga-PSMA-11, but its longer half-life combined with its superior energy characteristics and non-urinary excretion overcomes some practical limitations of ^68^Ga-labelled PSMA-targeted tracers.

**Electronic supplementary material:**

The online version of this article (doi:10.1007/s00259-016-3573-4) contains supplementary material, which is available to authorized users.

## Introduction

The introduction of the ^68^Ga-labelled prostate-specific membrane antigen (PSMA) targeted positron-emitting-tomography/computed-tomography (PET/CT) tracer Glu-urea-Lys(Ahx)-HBED-CC (PSMA-11) has proven highly sensitive for the detection of disseminated prostate cancer (PCa). In two studies (involving 319 patients and 248 patients, respectively) sites of biochemical recurrence (BCR) were localized in 90 % of patients including those with modest elevations in prostate specific antigen (PSA) [1, 2]. PSMA-11 PET/CT appears superior in sensitivity to other PET agents such as Choline-PET/CT [3, 4]. This high sensitivity could have significant clinical implications for modifications of treatment at various stages of prostate cancer ranging from initial diagnosis to treatment monitoring of castration resistant metastases. For instance, ^68^Ga-PSMA-11 PET/CT had a significant impact on radiotherapy planning, resulting in meaningful changes in treatment planning in over 50 % of patients [5, 6]. Although extensively studied in the recurrence and metastatic setting, there has been relatively little attention paid to the use of PSMA-PET in initial staging [7–10]. One challenge of ^68^Ga-PSMA-11 imaging is that the agent is rapidly excreted via the urinary tract resulting in intense accumulation in the bladder, thus, obscuring the prostate. This has resulted in the development of other agents with slower urinary excretion. One such candidate, ^99m^Tc-MIP-1404, has been chosen for evaluation in phase-2 (NCT01667536) and phase-3 (NCT02615067) studies in North America for primary staging of PCa [11]. However, ^99m^Tc is a single photon emitter and thus, has neither the sensitivity nor can gain the spatial resolution of PET-based agents. Another challenge for ^68^Ga-PSMA-11 PET/CT is the limited availability of the ^68^Ga via local radionuclide generators. Each generator provides only one or two elutions per day and is not only a substantial upfront investment but requires separate syntheses at different times of the day in a local radiopharmacy. Compared to ^18^F (0.65 MeV), the positron energy of ^68^Ga is higher (1.90 MeV), reducing the theoretical maximum spatial resolution [12]. Finally, the short half-life of ^68^Ga relative to ^18^F, (68 vs. 110 min) limits the ability to produce agents in a central facility and ship them to distributed imaging centers.

**Fig. 1:**
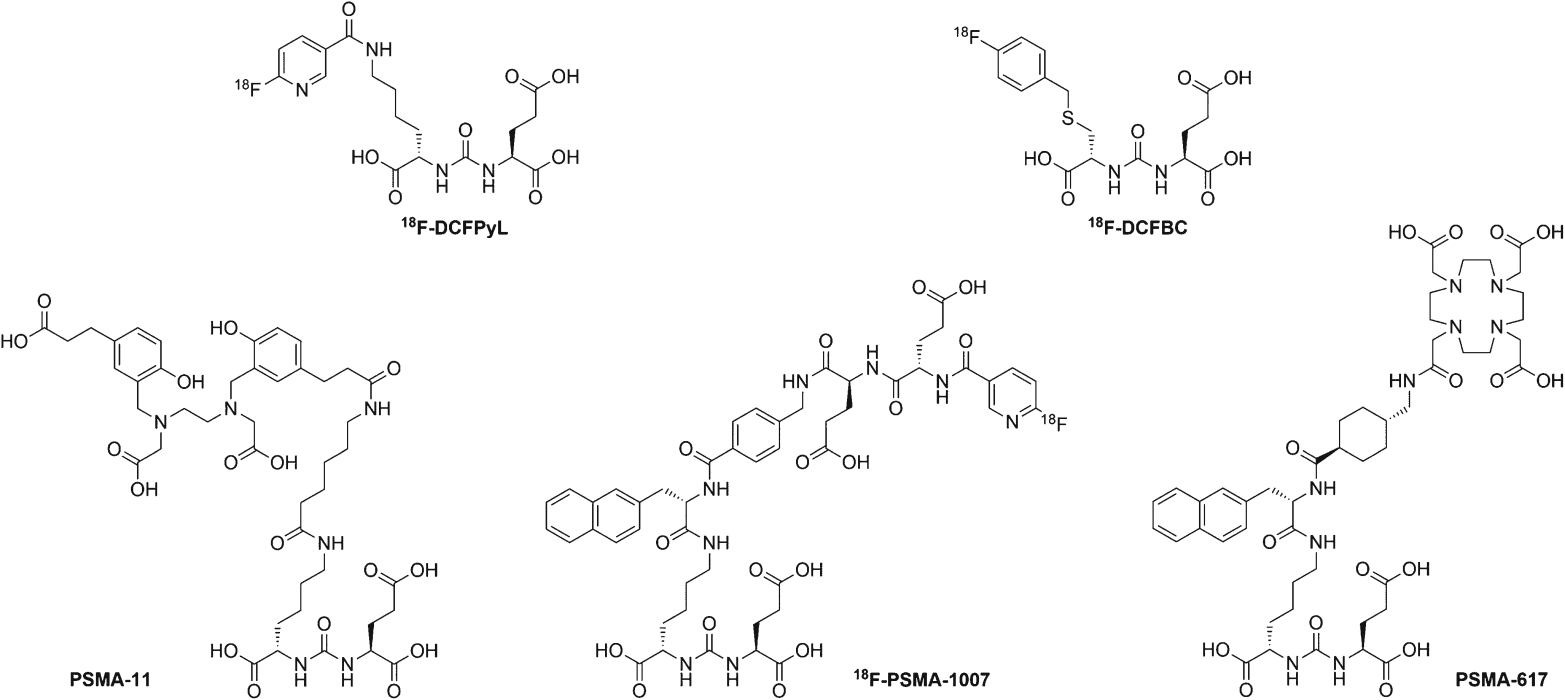
Comparison of different PSMA-ligands (^18^F-DCFBC, ^18^F-DCFPyL, ^68^Ga-PSMA-617, ^68^Ga-PSMA-11, ^18^F-PSMA-1007)

The first generation of ^18^F-labelled PSMA-ligands, such as ^18^F-DCFBC, suffered from high background due to slow blood clearance [13]. This has recently been addressed with the introduction of the second generation compound ^18^F-DCFPyL [14], a ligand which is characterized by fast elimination via the urinary route. However, neither ^18^F-DCFBC nor ^18^F-DCFPyL includes a chelator capable of binding therapeutic nuclides. PSMA-617 includes a chelator for labeling with diagnostic ^68^Ga as well as β-emitting ^177^Lu [15, 16] or α-emitting ^225^Ac [17]. Here, we present initial data on the biodistribution, radiation dosimetry and efficacy of ^18^F-PSMA-1007, a new ^18^F-labelled PSMA-ligand structurally related to PSMA-617 (Fig. 1) [18].

## Material and methods

### Synthesis and quality control of ^18^F-PSMA-1007

The non-radioactive reference compound ^19^F-PSMA-1007 and the PSMA-1007 precursor were synthesized using solid phase chemistry [19]. ^18^F-PSMA-1007 was produced on an automated synthesis module (Trasis AllInOne) in a two-step synthesis using the prosthetic group 6-[^18^F]F-Py-TFP [20] for coupling the PSMA-1007 precursor, and this was purified by semi-preparative HPLC. The detailed description of the radiolabelling process was published elsewhere [21]. A direct radiofluorination method is being developed. Radio-HPLC was performed to determine the chemical identity and the chemical and radiochemical purity of ^18^F-PSMA-1007. The chemical structure of ^18^F-PSMA-1007 is presented in Fig. 1. Residual solvents were determined by gas chromatography. Radionuclide purity was controlled by half-life measurement. Integrity of the sterile filter after filtration was assessed using the bubble-point test. The product solution was tested for sterility, bacterial endotoxins (LAL-test), pH, colorlessness and particles.

### PET/CT-imaging

All imaging was performed on a Biograph mCT Flow scanner (Siemens, Erlangen, Germany). PET was acquired in 3-D mode (matrix 200 × 200) using FlowMotion (Siemens). The emission data was corrected for randoms, scatter and decay. Reconstruction was performed with an ordered subset expectation maximization (OSEM) algorithm with two iterations/21 subsets and Gauss-filtered to a transaxial resolution of 5 mm at full-width at half-maximum (FWHM); Attenuation correction was performed using the unenhanced low-dose CT data. The CT-scans were reconstructed to a slice thickness of 5 mm, increment of 3–4 mm, soft tissue reconstruction kernel (B30), using CareDose (Siemens).

### Volunteers and patients

To determine initial dosimetry, three healthy volunteers (normal PSA) underwent ^18^F-PSMA-1007 PET/CT with scans obtained at multiple time points up to 6 h p.i. The healthy subjects were imaged in three blocks. Block 1 began at PET-1 (start 5 min p.i.) and extended to PET-7 (ending 140 min p.i.), block 2 began at PET-8 (start 180 min p.i.) and extended to PET-9 (240–270 min p.i.) and block 3 began at PET-10 (440–480 min p.i.). A non-enhanced low-dose CT (estimate 1.4 mSv, respectively) for attenuation correction was performed at the beginning of each block, followed by serial emission scans without moving the volunteers in between.

All patients (*n* = 10) gave written informed consent to receive ^18^F-PSMA-1007 following guidelines of the German Pharmaceuticals Act §13(2b). All patients had newly diagnosed high-risk prostate cancer (median PSA-level 14 ng/ml; range 5.8–87.3 ng/ml) with a median age of 65 years (range 55–77) and underwent ^18^F-PSMA-1007 PET/CT imaging 1 and 3 h post injection. The attenuation correction CTs were also performed 1 and 3 h post tracer injection. All men were treated clinically with a multimodal approach and eight men underwent radical prostatectomy with extended pelvic lymphadenectomy. Two patients who were found to have metastatic disease did not undergo prostatectomy. The data were analyzed retrospectively with approval of the local ethics committee. Detailed patient characteristics and final histopathological evaluation are provided in Table 1.

**Table 1:** Patient characteristics

Patient No.	Age (y)	^18^F-PSMA-1007 (MBq)	Gleason score	Initial PSA (ng/ml)	TNM-classification	PET/CT LN-metastases	PCa SUV_max_ (1 h p.i.)	PCa SUV_max_ (3 h p.i.)
1	77	356 MBq	9	40.0	pT3b, pN1 (4/41), L1, V0, Pn1	5	54.94	76.24
2	72	347 MBq	9	15.3	cT3b, cN1, cM1	7	47.71	74.11
3	55	315 MBq	9	14.0	pT3b, pN1 (5/40), L1, V0, Pn1	4	10.92	14.84
4	65	301 MBq	9	13.9	pT3b, pN1 (4/43), L0, V0, Pn1	4	24.30	30.18
5	64	331 MBq	9	10.0	pT3b, pN1 (3/48 LK), L1, V0, Pn1	3	18.85	27.49
6	64	240 MBq	7	12.2	pT3a, pN1 (3/57), L1, V1, Pn1	3	12.98	22.57
7	62	139 MBq	8	8.5	pT3a, pN0 (0/21), L0, V0, Pn1	0	15.53	27.45
8	69	319 MBq	8	5.8	pT3a, pN0 (0/32), L0, V0, Pn0	0	36.69	58.56
9	61	289 MBq	7	87.3	cT3, cN1, cM1	3	35.40	58.22
10	73	111 MBq	7	31.0	pT3a, pN0 (0/27), L0, V0, Pn1	0	16.31	19.61

### Radiation dosimetry

The dosimetry analysis was performed using the QDOSE dosimetry software suite (ABX-CRO, Germany). Automatic rigid co-registration and, if necessary, additional manual correction, were performed for all PET and CT data-sets. Kidneys, liver, spleen, whole heart, upper and lower large intestine, parotid glands, submandibular glands and urinary bladder were segmented into volumes of interest (VOIs) using a percentage of maximum threshold between 20 and 30 % and the corresponding CT as guidance. Afterwards the time activity curves (TACs) were calculated for all organs. The TACs for red marrow were derived from venous blood samples, and the red marrow dose was calculated as described previously [22, 23]. The urinary bladder TAC was a combination of estimated activity in the urinary bladder VOI in PET and measured activity of voided urine. Curve fitting was applied to all TACs according to the software. Kidneys, salivary glands, upper and lower large intestine and heart were fitted with a bi-exponential function. For liver, spleen and urinary bladder content, a mono-exponential fit to the last three time points was performed.

The cumulative activity Ã between time 0 and the first measured time point was calculated assuming a linear increase from 0 to the first measured activity. The Ã between the first measured time point and the last measured time point was integrated numerically using trapezoidal approximation. The Ã from the last measured time point to infinity was integrated using the fitted function. The total body Ã was calculated based on the injected activity assuming only physical decay neglecting the voided urine. The Ã of the remainder of the body was then automatically calculated by subtracting all source organs from the total body activity. All source organ residence times were calculated by dividing the Ã by the injected activity.

Absorbed and effective dose calculations were performed using the ICRP endorsed IDAC 1.0 package [24] which is integrated into QDOSE. In addition, residence times of all source organs and the remainder of the body were exported as an OLINDA case file for dose calculation in OLINDA 1.1 [25]. The absorbed doses to the salivary glands and prostate were determined using the spherical model [26]. The organ masses for the salivary glands were taken from ICRP publication 89 [27] with 25 g estimated weight for the parotid and 12.5 g for the submandibular gland.

### Biodistribution

During the dosimetry evaluation, urine was collected at the following intervals: 0–2 h, 2–4 h, and 4–6 h. Venous blood samples were obtained at 2, 5, 10, 15, 30, 45, 60, 80, 120, 180, 240, and 360 min post injection. After the whole blood was sampled, the remaining volume was centrifuged and serum was extracted. Serum activities were measured in a well counter and corrected for decay. Activity concentrations in blood vs. serum were compared using the individually determined (clinical routine lab) hematocrit. Total blood volume was estimated from size, weight, and hematocrit.

The tracer biodistribution in patients was quantified by SUV_mean_ and SUV_max_ at 1 h and 3 h post injection. For calculation of the standardized uptake value (SUV), circular regions of interest were drawn around the area with focally increased uptake in transaxial slices and automatically adapted to a three-dimensional VOI with e.soft software (Siemens) at a 40 % isocontour. Primary tumors and lymph node metastases were evaluated separately. The normal bladder (after voiding), background (pelvic fat), blood, brain, salivary and lacrimal glands, lung, liver, spleen, pancreas, small intestine, and kidneys were evaluated with a 2–3 cm sphere placed inside the organ parenchyma.

### Histopathological evaluation

Analyses of prostatectomy specimens were performed under the supervision of dedicated uropathologists, using International Society of Urological Pathology nomenclature [28]. Pathologists were blinded to the PET/CT results. In addition, representative sections were stained for immunohistochemistry. To accomplish this, sections were deparaffinized in xylene and rehydrated in a graded ethanol series. Antigen retrieval was performed with a steam cooker using retrieval buffer (Target Retrieval Solution, Dako). A mouse monoclonal antibody against PSMA (clone 3E6, Dako) was used at a 1:100 dilution and incubated overnight at 4 °C. Immunodetection was performed using the Histostain-Plus detection kit (Invitrogen) according to manufacturer’s recommendations. Stained sections were scanned using a Nanozoomer 2.0-HT Scansystem (Hamamatsu Photonics) to generate digital whole slide images.

## Results

### Adverse events

All patients and healthy volunteers tolerated the examination well. No drug-related pharmacological effects or physiologic responses occurred. All observed parameters (e.g., blood pressure, heart rate, body temperature) remained normal and unchanged during and after the examination. No patient reported subjective symptoms.

### Radiation dosimetry

**Fig. 2:**
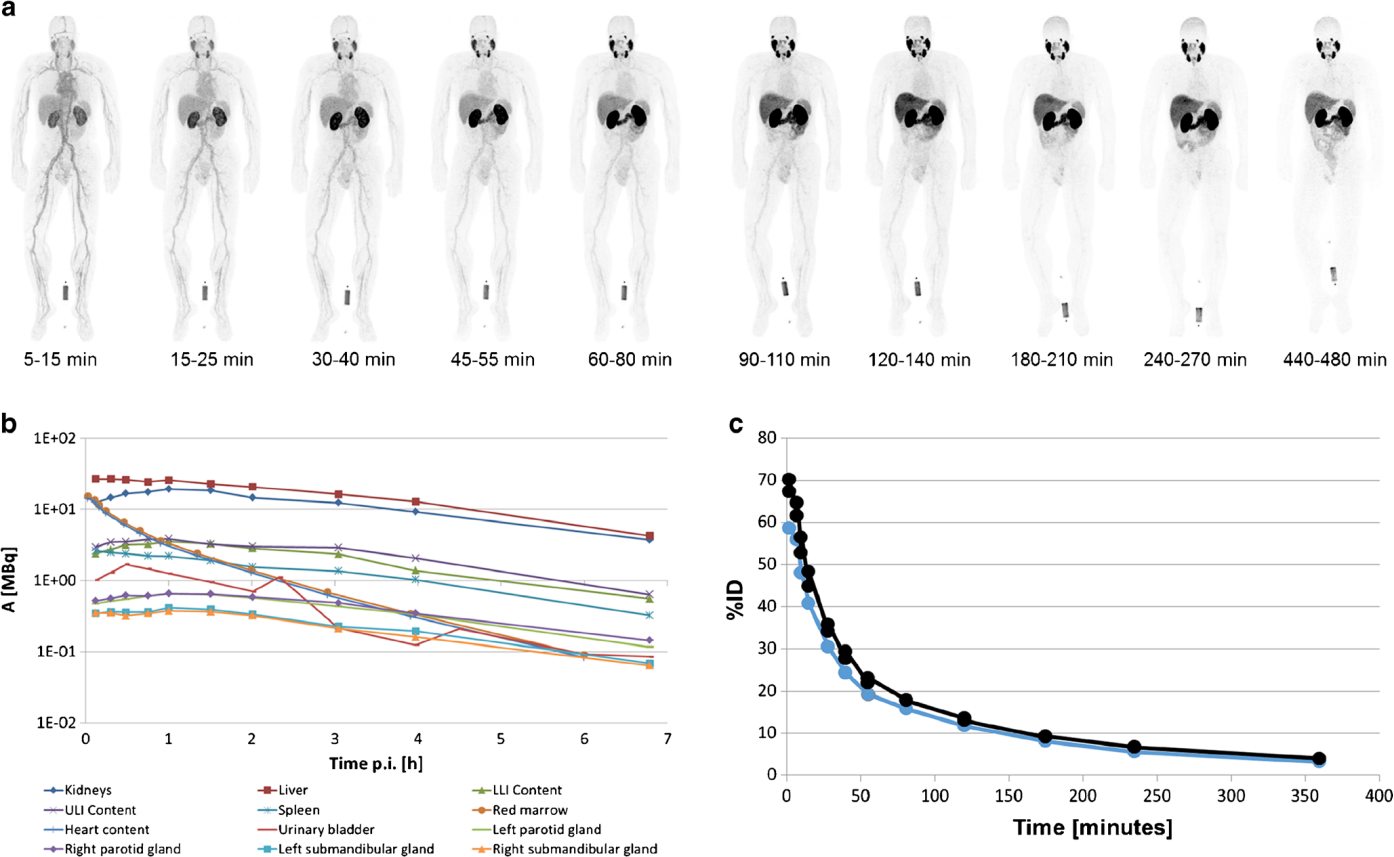
Maximum Intensity Projections (MIP) of ten serially performed (5 min–8 h p.i.) ^18^F-PSMA-1007 PET-scans in one healthy volunteer (**a**); biodistribution corrected for decay. Time-activity-curves of normal organs derived from PET volume-of-interest (**b**). Blood and serum time-activity-curves derived from serial blood-sampling, expressed as percent injected dose in a total blood volume of 6.1 l (**c**)

Maximum intensity projection (MIP) images of the serially performed whole-body PET-scans of one healthy volunteer are depicted in Fig. 2a and demonstrate distinct organ uptake for lacrimal glands, salivary glands, liver, spleen, small intestine and kidneys. The fitting of these organ VOIs and blood/plasma samples are presented in Fig. 2b and c. According to the average of three healthy volunteers, the radiation dosimetry revealed an effective dose of 0.022 mSv/MBq, i.e., 4.4–5.5 mSv for an injected dose of 200–250 MBq. Mean values of dedicated organ doses are presented in column 1 of Table 2. The individual calculations of each subject’s organ absorbed doses, effective half-lives and residence times are provided as supplemental data. As a reference, Table 2 also presents the corresponding organ absorbed doses for the other ^18^F-labelled PSMA-ligands DCFPyL (column 5), DCFBC (column 6), the ^68^Ga-labelled compounds PSMA-11 (columns 2,3) and PSMA-617 (column 4). In addition, the mean absorbed doses for the submandibular glands were 0.075 mGy/MBq, parotid glands 0.09 mGy/MBq and prostate gland 0.045 mGy/MBq. The intra-individual dose ratios are very similar for all PSMA-targeted agents; in comparison to ^18^F-DCFPyl, the effective dose of ^18^F-PSMA-1007 is slightly higher, while lower clearance through the urinary route reduces dose to the bladder wall.

**Table 2:** Dosimetry (OLINDA) comparison of ^18^F-PSMA-1007 with other PSMA-targeted tracers

Absorbed Dose (mGy/MBq)						
Organ	^18^F-PSMA-1007	^68^Ga-PSMA-11	^68^Ga-PSMA-11	^68^Ga-PSMA-617	^18^F-DCFPyL	^18^F-DCFBC
	This work	Afshar-Oromieh et al. [32]	Pfob et al. [31]	Afshar-Oromieh et al. [30]	Szabo et al. [14]	Cho et al. [13]
Adrenals	1.94E–02	1.42E–02	4.67E–03	1.48E–02	3.11E–02	1.85E–02
Brain	7.20E–03	9.00E–03	1.21E–03	3.53E–02	2.19E–03	4.21E–03
Breast	8.06E–03	8.80E–03	2.10E–03	1.03E–02	4.57E–03	8.51E–03
Gallbladder wall	2.22E–02	1.44E–02	4.23E–03	1.50E–02	1.44E–02	1.79E–02
Lower colon	4.83E–02	1.23E–02	4.64E–03	1.33E–02	1.05E–02	2.47E–02
Small intestine	1.56E–02	1.63E–02	3.64E–03	1.83E–02	9.13E–03	2.36E–02
Stomach	1.42E–02	1.20E–02	3.02E–03	1.30E–02	1.16E–02	3.02E–02
Upper colon	4.08E–02	5.40E–02	3.42E–03	4.48E–02	1.67E–02	2.34E–02
Heart wall	2.51E–02	1.09E–02	2.78E–03	1.20E–02	1.29E–02	2.92E–02
Kidneys	1.70E–01	2.62E–01	1.21E–01	2.06E–01	9.45E–02	2.84E–02
Liver	6.02E–02	3.09E–02	2.07E–02	2.88E–02	3.80E–02	2.46E–02
Lungs	1.11E–02	1.02E–02	7.89E–03	1.15E–02	1.08E–02	2.45E–02
Muscle	1.00E–02	1.05E–02	1.61E–03	1.15E–02	6.32E–03	9.69E–03
Pancreas	1.92E–02	1.38E–02	4.08E–03	1.45E–02	2.44E–02	1.92E–02
Red marrow	1.33E–02	9.20E–03	8.06E–03	1.00E–02	1.04E–02	1.70E–02
Osteogenic cells	1.55E–02	1.42E–02	6.77E–03	5.40E–02	9.58E–03	1.82E–02
Skin	7.30E–03	8.85E–02	2.09E–03	9.50E–03	4.05E–03	7.30E–03
Spleen	7.39E–02	4.46E–02	4.13E–02	2.85E–02	1.85E–02	1.72E–02
Testes	8.37E–03	1.04E–02	3.43E–03	1.15E–02	1.01E–02	1.54E–02
Thymus	9.90E–03	9.90E–03	2.22E–03	1.15E–02	5.56E–03	1.10E–02
Thyroid	8.50E–03	9.70E–03	2.14E–03	1.13E–02	8.56E–03	1.17E–02
Urinary bladder wall	1.87E–02	1.30E–01	1.64E–01	9.03E–02	8.64E–02	3.24E–02
ED (mSv/MBq)	2.20E–02	2.36E–02	1.58E–02	2.08E–02	1.39E–02	1.99E–02

### Normal-organ Biodistribution and tumor uptake

**Fig. 3:**
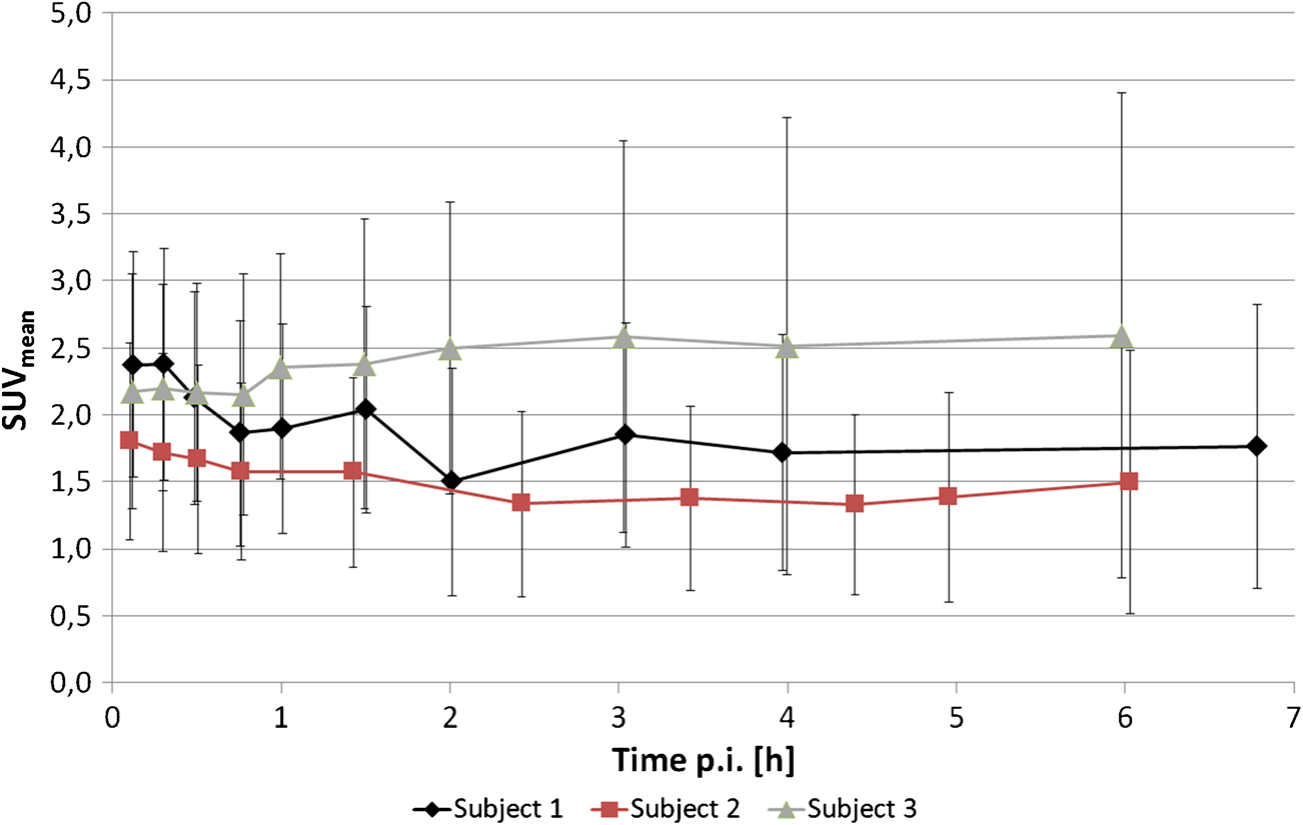
Time-activity-curves of ^18^F-PSMA-1007 from a volume-of-interest covering the healthy prostate from three volunteers (SUV_mean_ with standard deviations)

In healthy subjects, the blood pool contained mean 76, 22, 12 and 8 % of the injected dose at 2 min, 1 h, 2 h and 3 h p.i., respectively. Approximately 95 % of the blood pool activity was found in the serum; the blood and serum curves converge with more complete clearance. It is possible that there was a minimal and reversible diffusion of the agent into blood cells; however, irreversible binding to blood cells can be safely excluded. Clearance via the urinary tract was minimal and in average only 1.2, 0.7 and 0.5 % of the injected activity were eliminated in the urine during the 0–2, 2–4 and 4–6 h intervals, respectively. In one healthy volunteer, symmetric tracer uptake (SUV_mean_ 2, perfusion dependent background <1) in the axillary lymph nodes was observed. Taking into account his normal PSA-value and an increased CRP and BSR (blood sedimentation rate), this finding implies the potential of false-positive uptake in inflammatory activated lymph nodes. The time-activity-curve of the normal prostate tissue in the healthy volunteers is provided in Fig. 3 and may serve as a reference for future dynamic studies of primary prostate cancer.

**Fig. 4:**
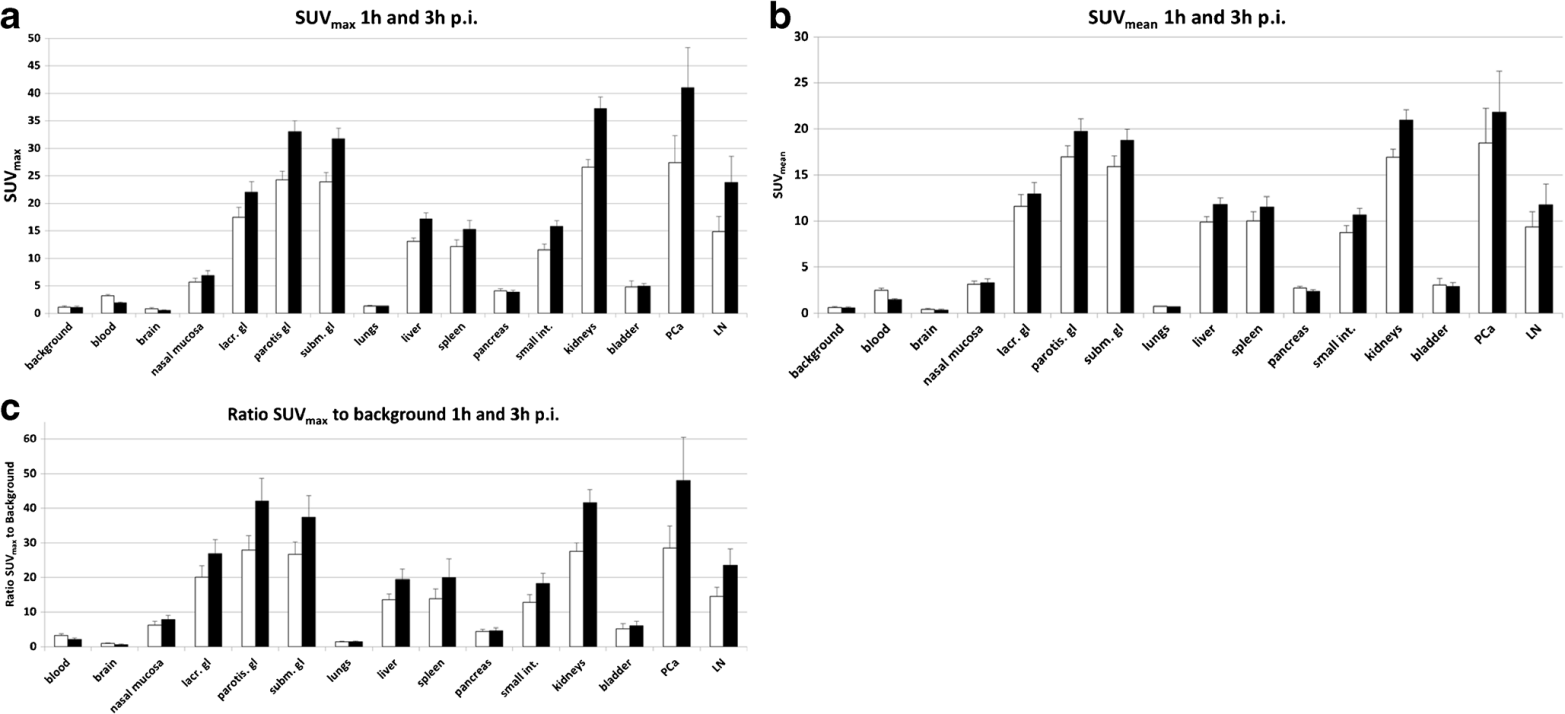
**a:** Biodistribution of mean SUV_max_ of ^18^F-PSMA-1007 in normal organs (blood, brain, nasal mucosa, lacrimal gland, parotid and submandibularis glands, lungs, liver, spleen, pancreas, small intestines, kidneys and bladder) and tumor lesions (prostate cancer (PCa) and lymph node (LN) metastases) with its standard error. **b:** Biodistribution of mean SUV_mean_ of ^18^F-PSMA-1007 in normal organs (blood, brain, nasal mucosa, lacrimal, parotid and submandibularis glands, lungs, liver, spleen, pancreas, small intestines, kidneys and bladder) and tumor lesions (prostate cancer (PCa) and lymph node (LN) metastases) with its standard error. **c:** Biodistribution of mean SUV_max_ to background of ^18^F-PSMA-1007 in normal organs (blood, brain, nasal mucosa, lacrimal, parotid and, submandibular glands, lungs, liver, spleen, pancreas, small intestines, kidneys and bladder) and tumor lesions (prostate cancer (PCa) and lymph node (LN) metastases) with its standard error

**Fig. 5:**
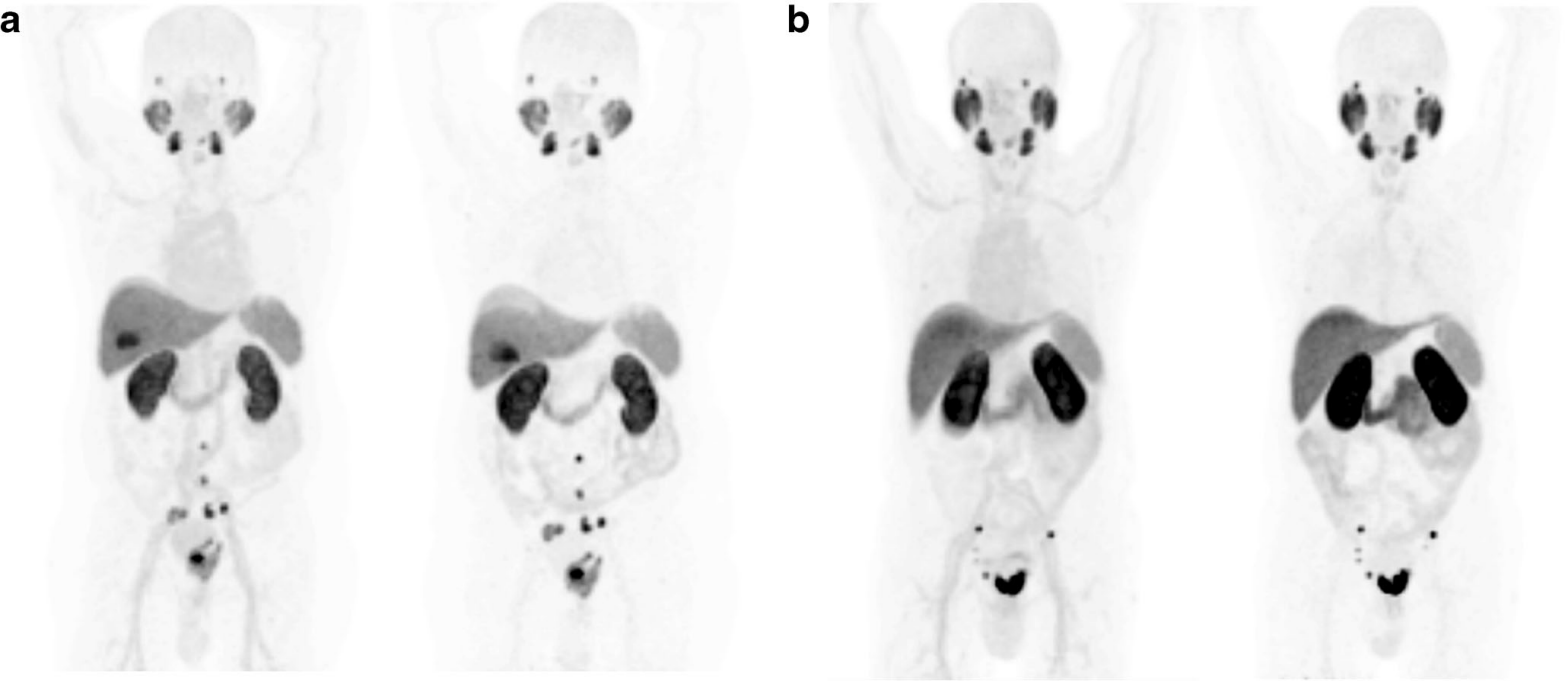
**a:** Patient 2, a 72-year-old patient (PSA 15 ng/ml) diagnosed with Gleason 9 (5 + 4) prostate cancer. Patient presented with a large tumor mass in the prostate gland infiltrating the left seminal vesicle and metastases to several lymph nodes in the pelvis. Two metastatic lymph nodes are located outside the pelvis, both paraaortic at level L3/4 and L5. ^18^F-PSMA-1007 shows high tumor uptake after 1 h and 3 h p.i in the maximum intensity projection PET-scan. Due to the lipophilic characteristics of ^18^F-PSMA-1007, the hepatobiliary clearance can be observed while urinary excretion is minimal. **b:** Patient 1, a 77-year-old prostate cancer patient (PSA 40 ng/ml) shows a large tumor mass on the mid and apical prostate and several lymph node metastases

**Fig. 6:**
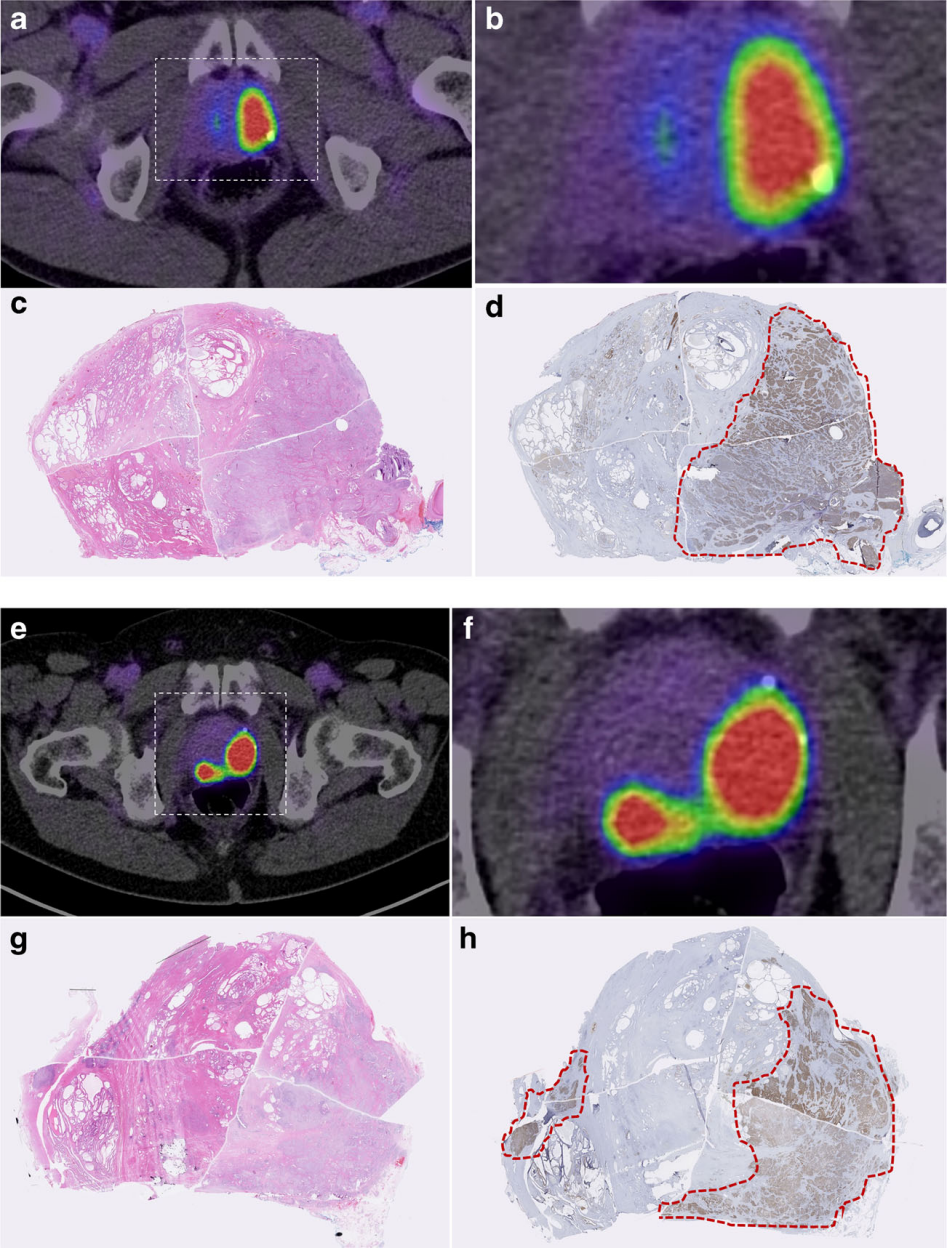
Comparison of virtual whole mount histopathology (H&E and PSMA-immunostaining) and PSMA PET-findings. Transaxial PET/CT-scan of patient 1 (**a**, **b**, **e**, **f**) and corresponding histopathology of the subsequent prostatectomy specimen; H&E staining (**c**, **g**); PSMA-immunostaining with outlined tumor contours in red (**d**, **h**)

The uptakes in the primary tumor, lymph node metastases and normal-organ biodistribution at 1 h and 3 h p.i. in the ten patients with prostate cancer are presented in Fig. 4. The mean tumor-uptake was 5- and 10-fold higher at 1 h and 3 h p.i., compared to intra-vesical urine. The primary tumor was readily seen on PET-imaging in Fig. 5. The intra-prostatic peak uptake correlated strongly with the intraprostatic tumor mass as shown in Fig. 6. Between 1 h and 3 h p.i. the absolute tumor uptake demonstrated an increase in SUV_max_ and SUV_mean_ by around 50 %. Simultaneously, the blood-pool declined by around −40 %. Thus, the delineation of tumors improved at the later time point at 3 h p.i. (Fig. 5) reaching an average SUV_max_ of 41 in the primary tumors.

### Correlation of imaging and post-surgery histopathology

The primary tumors and lymph node metastases were first diagnosed on PET. Within a median of 20 days, eight patients underwent radical prostatectomy and extended pelvic lymphadenectomy (Table 1). Two patients had metastatic disease at initial diagnosis and did not undergo surgery. In total 309 pelvic lymph nodes were histologically examined, out of which 290 were negative and 19 were positive (median tumor diameter 5 mm; range 1–18 mm). ^18^F-PSMA-1007 PET/CT demonstrated excellent sensitivity (94.7 %). Overall, ^18^F-PSMA-1007 PET/CT detected 18 of 19 lymph node metastases in the pelvis including a node with a diameter as small as 1 mm. In contrast to one healthy subject who was suspicious for unspecific uptake in inflammatory lymph nodes, there were no false-positive findings in the patient cohort (specificity 100 %). Similarly, the peak intra-prostatic uptake correlated with the main tumor mass (Fig. 6). Positive PET-findings outside of the pelvis were not histopathologically validated.

## Discussion

In this study we evaluated the radiation dosimetry, biodistribution and preliminary efficacy of the new tracer, ^18^F-PSMA-1007.

Compared to other similar agents, the effective dose of ^18^F-PSMA-1007 PET/CT (4.4–5.5 mSv for 200–250 MBq) was comparable (Table 2). The effective organ half-life and, hence, the exposure, depends primarily on the short physical half-life of ^18^F rather than the biological half-life of the carrier molecule. All Glu-urea-based PSMA-targeted tracers share a similar physiological tracer distribution, thus the organ ratios differ only minimally among the various ligands. One strength of our dosimetry estimation is that we utilized a large number of ten head-to-toe whole-body scans performed up to 6 h p.i. (i.e. > 3 physical half-lives of ^18^F) allowing for excellent curve fitting. One source of uncertainty is the limited number of healthy subjects (*n* = 3). Nonetheless, since the normal organ biodistribution of ^18^F-PSMA-1007 has proven highly similar to other PSMA-targeted radiotracers, such as PSMA-11, PSMA-617, DCFBC and DCFPyL [13, 14, 29, 30], we consider that the results of this first dosimetry study are in keeping with prior results. The performance of dosimetry in normal volunteers is preferred to performing it in patients with large tumor masses as these could cause tumor “sink effects” distorting the normal biodistribution. This has proven to be less of a concern in PSMA ligands such as ^18^F-DCFPyL, in which it was shown that the dosimetry in normal organs was reliably assessed independently of the presence of tumor [14].

The average 1 h and 3 h SUV_max_ of ^18^F-PSMA-1007 in tumor tissue, parotid glands, liver, kidneys and spleen can be compared to data for PSMA-11 [9, 29] and PSMA-617 [30], because the evaluation of these ^68^Ga-labelled PSMA-ligands were also performed at our institution with similar imaging protocols, cross-calibrated scanners and analysis software. In contrast, ^18^F-DCFBC and ^18^F-DCFPyL have been evaluated with different scanners and imaging protocols which makes the comparison less accurate. However, even the comparison with PSMA-11 and PSMA-617 has limitations. The low patient numbers might introduce errors, especially on the highly variable inter-individual tumor uptake. In addition, due to the higher positron energy and increased spill-out-effects, ^68^Ga quantification might be systematically underestimated in small lesions. Furthermore, the patient selection differs: PSMA-617 was evaluated in a heterogeneous group of patients [30], whereas PSMA-11 was evaluated primarily in BCR patients [29]. In contrast PSMA-1007 is now evaluated in patients with treatment-naive high-risk PCa. Therefore, quantification per SUV should be interpreted cautiously. Semi-quantitatively the ratio tumor-to-parotid is comparable for all tracers. PSMA-617 has the lowest uptake in liver, kidney and spleen, which was a goal when developing theranostic PSMA-ligands for radionuclide therapy. However, as known from ^177^Lu-PSMA-617 dosimetry [15], PSMA-617 has slower tumor accumulation than PSMA-11 and is considered suboptimal when used as ^68^Ga-labelled PET-tracer. ^18^F-PSMA-1007 shares the slightly slower tracer kinetics of PSMA-617, although this is less crucial due to the longer half-life of ^18^F in comparison to ^68^Ga. Due to its structural similarity to PSMA-617, it has been suggested that it could be an ideal diagnostic surrogate for patients considering ^177^Lu-PSMA-617 therapy [18]. For a pure diagnostic tracer the slightly higher uptake of ^18^F-PSMA-1007 in visceral organs is negligible with regard to radiation burden and is also of limited clinical impact as visceral metastases occur late in the course of the disease. More importantly the increased uptake in tumor tissue compared to other tracers improves tumor-to-background ratios making it easier to detect small lymph node metastases. It remains to be seen whether patients with advanced castration-resistant PCa will have higher liver background which may interfere with the detection of metastases in comparison to the actual reference compound ^68^Ga-PSMA-11. One clear advantage for local staging is that ^18^F-PSMA-1007 is temporarily retained in the kidney parenchyma. For ^18^F-PSMA-1007, clearance via the urinary tract was only 1.2 % injected activity during 0–2 h and another 0.7 % 2–4 h p.i.; in comparison, 11 % of ^18^F-DCFPyl is eliminated during the first 2 h via the urinary tract and another 5 % until 3 h p.i. [14]. Bladder content of up to SUV_max_ 40 (^68^Ga-PSMA-617) and SUV_max_ 100 (^68^Ga-PSMA-11) has been reported for the ^68^Ga-containing compounds, respectively [29–32]. In contrast, the content of the urinary bladder was SUV_max_ 5 for ^18^F-PSMA-1007 (Fig. 4). Obviously, this agent demonstrates delayed urinary excretion and thus fulfills some of the design criteria for the ideal PSMA-targeted PET tracer. This also explains the lower radiation dose to the urinary bladder wall in comparison to other PSMA-tracers, while kidney dose is comparable (Table 2).

In the evaluation of first patients, the sensitivity of ^18^F-PSMA-1007 for small lymph node metastases was approximately 95 %. Metastatic nodes as small as 1 mm in diameter were discovered. In a retrospective analysis of patients who received pelvic lymphadenectomy after imaging with a variety of PSMA-targeted tracers, the sensitivity for these very small nodes was limited [33]. Thus, small lesion detection is a very promising early result for the new compound. However, the mentioned study suffered from several limitations, including a long interval between imaging and surgery or the lack of standardization in regard to imaging protocols and documentation of findings [33]. Therefore, it cannot be concluded that the higher sensitivity in our cohort is caused solely by the improved tracer. Nevertheless, lymph node metastases with median diameters of 5 mm are close to the technical resolution limits of PET with ^68^Ga-PSMA tracers and, therefore, it would be reasonable that ^18^F-PSMA tracers might perform at a higher level.

## Conclusion

^18^F-PSMA-1007 is an attractive alternative to ^68^Ga-PSMA-11 and ^18^F-DCFPyL. Radiation burden of a 200–250 MBq injection translates to 4.4–5.5 mSv effective dose, similar to other established PET tracers. ^18^F-PSMA-1007 can be produced in large amounts per batch in PET radiopharmacies with an on-site cyclotron, reducing the demand for multiple tracer syntheses per day and enabling transfer to satellite centers. Preliminary data suggest reduced urinary excretion and high tumor to background ratios contribute to exceptional sensitivities including for tiny tumor deposits in the body. Larger trials with this PET tracer are expected to further define its capabilities and role in the management of prostate cancer.

## Electronic supplementary material

Below is the link to the electronic supplementary material.

ESM 1 (PDF 241 kb)
