# Supplementary material for: F-18 labelled PSMA-1007: biodistribution, radiation dosimetry and histopathological validation of tumor lesions in prostate cancer patients
Source: Eur J Nucl Med Mol Imaging. 2016 Nov 26;44(4):678–88. doi: 10.1007/s00259-016-3573-4 (PMC5323462; doi:10.1007/s00259-016-3573-4)
Supplement: Supplementary file 1 — (PDF 241 kb) [file 259_2016_3573_MOESM1_ESM.pdf]

Effective Half Lifes

	Subject-1			Subject-2			Subject-3	
Organ	Eff1	Eff2		Eff1	Eff2		Eff1	Eff2
Heart Content	2.21E-01	9.81E-01		8.40E-02	5.68E-01		2.01E-01	8.54E-01
Heart Wall	NA	NA		NA	NA		NA	NA
Kidneys	2.17E+00	4.78E-01		5.82E-01	1.85E+00		1.89E+00	5.66E-01
LKidney	2.17E+00	5.15E-01		5.94E-01	1.85E+00		1.88E+00	5.94E-01
LLI Content	1.67E+00	6.41E-01		1.31E-01	2.13E+00		1.77E+00	2.35E-01
LParotidGland	8.89E-01	1.59E+00		1.68E+00	8.35E-01		7.03E-01	1.71E+00
LSubmandibularGland	1.63E+00	8.47E-01		1.70E+00	4.85E-01		3.40E-01	2.16E+00
Liver	2.06E+00	NA		1.78E+00	6.72E-01		1.86E+00	7.89E-01
Prostate	6.30E-01	2.15E+00		7.22E-01	2.00E+00		4.52E-01	2.31E+00
RKidney	2.16E+00	4.44E-01		5.70E-01	1.86E+00		5.43E-01	1.88E+00
RParotidGland	1.75E+00	9.47E-01		5.98E-01	1.82E+00		2.07E+00	4.94E-01
RSubmandibularGland	1.23E+00	1.23E+00		8.56E-01	1.56E+00		8.65E-01	1.60E+00
Red Marrow	2.22E-01	9.87E-01		8.38E-02	5.68E-01		2.01E-01	8.54E-01
Spleen	1.91E+00	NA		5.11E-01	1.86E+00		9.15E-01	1.88E+00
Total Body	NA	NA		NA	NA		NA	NA
ULI Content	2.00E+00	8.02E-01		1.29E+00	1.29E+00		2.42E+00	NA
Urinary Bladder Content	1.52E+00	NA		1.79E+00	NA		2.49E+00	NA
Whole Heart	2.54E-01	1.21E+00		2.66E-01	1.21E+00		2.90E-01	1.18E+00

Residence Times

Organ	Residence Time, Subject1 [MBq*h/MBq]	Residence Time, Subject2 [MBq*h/MBq]	Residence Time, Subject3 [MBq*h/MBq]
Heart Content	3.94E-02	3.78E-02	3.38E-02
Heart Wall	1.83E-02	1.69E-02	1.52E-02
Kidneys	3.22E-01	2.38E-01	2.32E-01
LKidney	1.75E-01	1.21E-01	1.14E-01
LLI Content	5.36E-02	6.68E-02	7.27E-02
LParotidGland	1.09E-02	1.63E-02	1.14E-02
LSubmandibularGland	6.44E-03	5.94E-03	6.04E-03
Liver	4.39E-01	4.49E-01	4.39E-01
Prostate	4.82E-03	5.34E-03	4.28E-03
RKidney	1.47E-01	1.17E-01	1.18E-01
RParotidGland	1.18E-02	2.00E-02	1.20E-02
RSubmandibularGland	5.81E-03	6.13E-03	5.49E-03
Red Marrow	4.26E-02	4.07E-02	3.64E-02
Spleen	3.59E-02	5.95E-02	9.33E-02
Total Body	2.64E+00	2.64E+00	2.64E+00
ULI Content	6.74E-02	6.76E-02	8.25E-02
Urinary Bladder	1.39E-02	2.45E-02	1.67E-02
Whole Heart	5.77E-02	5.47E-02	4.90E-02

IDAC Dose Calculation (Safety Dosimetry – Phantom: Adult)

Organ	NormalisedDose Subject-1 [mGy/MBq]	NormalisedDose Subject-2 [mGy/MBq]	NormalisedDose Subject-3 [mGy/MBq]
Adrenals	1,88E-02	1,83E-02	1,81E-02
Bladder	1,37E-02	1,57E-02	1,94E-02
Bone surfaces	9,66E-03	9,88E-03	9,99E-03
Brain	5,69E-03	6,16E-03	6,14E-03
Breast	6,79E-03	7,18E-03	7,18E-03
Gall bladder	2,12E-02	2,13E-02	2,12E-02
Stomach	1,23E-02	1,35E-02	1,28E-02
Small intestine	1,37E-02	1,48E-02	1,42E-02
Upper large intestine	3,70E-02	4,33E-02	3,73E-02
Lower large intestine	3,98E-02	5,20E-02	4,84E-02
Heart	2,51E-02	2,21E-02	2,40E-02
Kidneys	2,10E-01	1,55E-01	1,58E-01
Liver	6,14E-02	6,11E-02	6,23E-02
Lungs	9,65E-03	1,01E-02	1,01E-02
Muscles	8,50E-03	9,01E-03	8,92E-03
Oesophagus	8,39E-03	8,72E-03	8,81E-03
Ovaries	1,20E-02	1,34E-02	1,30E-02
Pancreas	1,74E-02	1,88E-02	1,78E-02
Red marrow	1,17E-02	1,16E-02	1,18E-02
Skin	6,08E-03	6,47E-03	6,43E-03
Spleen	4,81E-02	1,07E-01	7,17E-02
Testes	6,64E-03	7,26E-03	7,28E-03
Thymus	8,39E-03	8,72E-03	8,81E-03
Thyroid	6,76E-03	7,29E-03	7,27E-03
Remaining organs	8,83E-03	9,71E-03	9,39E-03
ED ICRP 60	2,15E-02	2,18E-02	2,14E-02
